# Restoring Function and Esthetics in Complete Edentulism: A Case Report of Implant-Supported Hybrid Denture

**DOI:** 10.7759/cureus.71399

**Published:** 2024-10-13

**Authors:** Ramanjeet K Grover, Shashikala Jain, Rajat Sharma, Priyanka Borse, Abhijeet Singh

**Affiliations:** 1 Department of Prosthodontics, Maharaja Ganga Singh Dental College and Research Centre, Sri Ganganagar, IND; 2 Department of Orthodontics, Maharaja Ganga Singh Dental College and Research Centre, Sri Ganganagar, IND

**Keywords:** dental implants, edentulous, esthetics, hybrid, immediate

## Abstract

Complete edentulism significantly affects the esthetics, function, and quality of life of the elderly. Implant-supported hybrid dentures are superior to conventional removable prostheses in terms of stability, esthetics, and masticatory function. This case report details the dental rehabilitation of a 75-year-old female patient seeking total dental restoration. Clinical evaluation revealed partial edentulism in both arches accompanied by residual root stumps. Eight endosseous implants were placed in the maxilla and seven in the mandible following a delayed loading protocol to ensure optimal osseointegration. A metal framework was fabricated, and occlusal adjustments were made to achieve a passive fit for the hybrid prosthesis. Post-rehabilitation care included educating the patient on oral hygiene and scheduling routine follow-up. The patient experienced marked improvements in speech, mastication, and esthetics, indicating successful rehabilitation. This case underscores the effectiveness of implant-supported hybrid dentures in delivering lasting functional and aesthetic improvements to edentulous patients, consistent with the current literature favoring their use over traditional removable prostheses.

## Introduction

Complete edentulism, or the absence of all teeth in one or both arches, remains a prevalent issue affecting millions of people worldwide, particularly in the aging population. Traditionally, removable complete dentures have been the primary solution for restoring the form, function, and esthetics of edentulous patients [1]. However, these prostheses often face challenges, such as instability, lack of retention, discomfort, and difficulty in maintaining proper nutrition and speech functions, and have emerged as a viable and superior option to address the limitations of conventional dentures, significantly improving the quality of life of edentulous patients [2]. In cases of pronounced alveolar ridge resorption or atrophic maxilla, pneumatization is often extensive, and the axial placement of implants in the posterior maxilla is difficult. In such cases, tilted implants with the use of transmucosal abutments come to be rescued such that an implant-supported hybrid prosthesis emerges as a superior option for rehabilitating completely edentulous patients.

Hybrid implant dentures are one such option that is particularly popular for rehabilitating completely edentulous patients. These dentures are fixed full-arch prostheses, often consisting of a titanium or cobalt-chromium framework veneered with acrylic or composite material [3]. They are supported by multiple implants, which improve retention, stability, and load distribution during mastication. Hybrid dentures offer significant advantages over traditional removable dentures, such as improved function, esthetics, patient comfort, and long-term bone preservation [4].

Hybrid implant-supported prostheses are particularly advantageous for patients exhibiting high smile lines or those with elevated esthetic expectations, as they seamlessly blend functional stability with naturalistic visual appeal. Additionally, the reduced weight and inherent flexibility of hybrid prostheses, compared to conventional metal-ceramic restorations, facilitate their application in scenarios where intra-arch space is limited without compromising structural integrity or longevity. A thorough clinical assessment of these multidimensional factors is indispensable for optimizing both the functional efficacy and esthetic outcome of implant-supported rehabilitations [5,6]. Moreover, hybrid dentures are fixed in the mouth, eliminating the discomfort associated with removable dentures, such as soreness from denture movement, the need for adhesives, and speech challenges [5,6].

However, hybrid implant-supported dentures are not without their drawbacks; one significant disadvantage is their higher cost compared to removable dentures, as the placement of multiple implants and the fabrication of a fixed prosthesis involve greater complexity and materials [7]. Additionally, the procedure requires adequate bone volume for implant placement; in some cases, bone grafting may be necessary to achieve optimal results [8]. Hybrid prostheses also require diligent oral hygiene practices to maintain peri-implant tissue health, as implant failure due to peri-implantitis can be a concern [9]. Finally, prosthetic maintenance of hybrid dentures can be more demanding than that of removable dentures, with occasional removal for professional cleaning or repairs [7].

Despite these disadvantages, hybrid dentures remain a favorable option for many edentulous patients. Studies have shown high survival rates for implants supporting hybrid dentures, with consistently high patient satisfaction due to improvements in comfort, esthetics, and oral function [2,5]. The "All-on-4" technique, which utilizes four implants strategically placed in the anterior mandible or maxilla, is a popular approach that minimizes surgical intervention while providing stable support for the prosthesis [10]. This technique often allows for immediate loading of implants, further improving patient convenience and reducing the treatment time [11].

In this case report, the rehabilitation of a completely edentulous patient using a hybrid implant-supported prosthesis which is fully fabricated utilizing computer-aided design/computer-aided manufacturing (CAD/CAM) technology, as polymethylmethacrylate (PMMA) typically adheres through mechanical interlocking, which is prone to deboning under persistent occlusal forces. The approach, advantages, and challenges are discussed, highlighting the efficacy of this treatment modality in providing a long-term functional and aesthetic solution for edentulous patients.

## Case presentation

**Figure 1:**
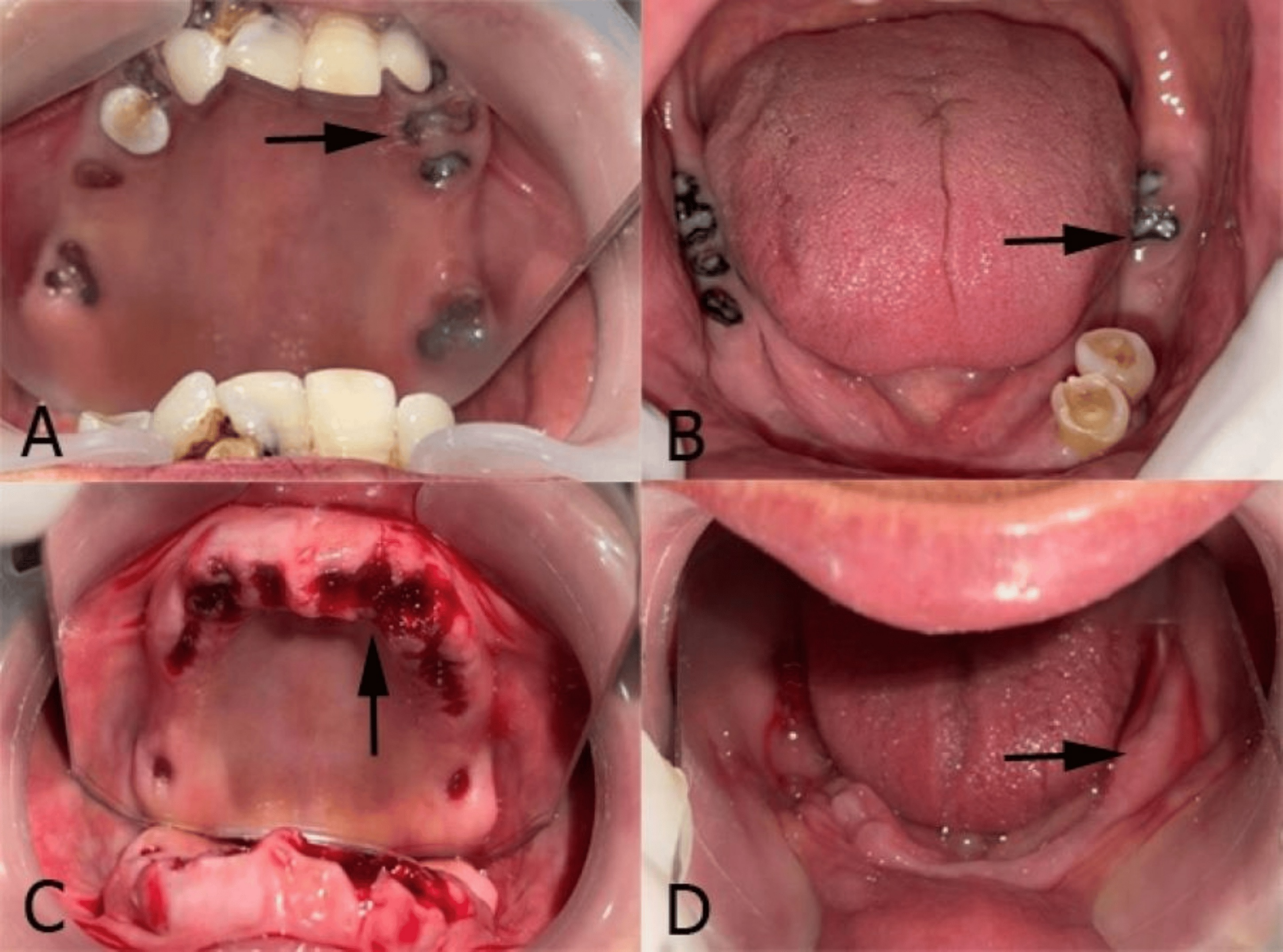
(A) Maxillary teeth and root stumps; (B) mandibular teeth and root stumps; (C) extraction of maxillary teeth and root stumps; (D) extraction of mandibular teeth and root stumps.

A 75-year-old partially edentulous female patient presented to the Department of Prosthodontics, Maharaja Ganga Singh Dental College and Research Centre, Sriganganagar, in May 2023, with the primary concern of missing teeth for which she wanted a replacement to improve esthetics and masticatory function. Clinical examination revealed partially edentulous maxillary and mandibular arches with residual root stumps. The patient expressed a desire for complete dental rehabilitation using a fixed prosthetic solution. The patient was informed about the treatment, and written consent was obtained. The preliminary examination began with a detailed medical and dental history to identify any systemic conditions or medications that may affect healing or the success of the procedure, but no relevant medical history was revealed. A thorough clinical examination was performed to assess the condition of the oral tissues, residual teeth, and root stumps (Figure 1).

An orthopantomogram (OPG) was used to evaluate bone quality and proximity to critical anatomical structures. Occlusal analysis was performed. Oral hygiene instructions were provided, and the necessary extractions for all root stumps and decayed teeth were completed to prepare the site for implants (Figure 1).

Surgical phase

**Figure 2:**
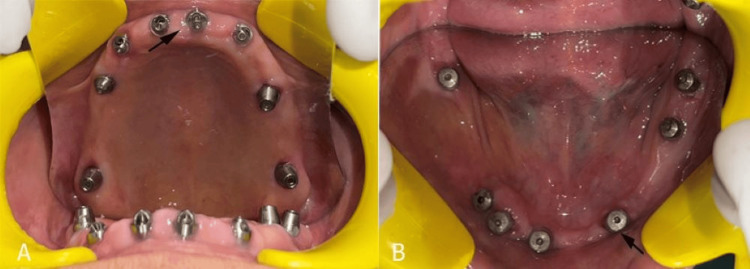
(A) Eight dental implants in the maxilla; (B) seven dental implants in the mandible.

The patient was administered prophylactic antibiotics before the surgical procedure. Amoxicillin + Clavulanic Acid (875 mg/125 mg) as a single dose was given one hour before the procedure to reduce the bacterial load during the surgery. Amoxicillin + Clavulanic Acid (500 mg/125 mg) every eight hours (three times a day) for five days post-surgery was given. Following extraction of the remaining teeth, extraction sockets were carefully debrided, and flapless immediate implant placement was performed under local anesthesia. Eight endosseous implants (Adin, Nagpur, India) were placed in the maxillary arch and seven implants in the mandibular arch using surgical templates with insertion torque values of 50 Ncm (Figure 2).

Primary stability was confirmed, and cover screws were placed on the implants. A delayed loading protocol of four months was followed in this case [3]. Provisional prosthetic devices lined with a soft material were constructed prior to the surgical intervention and secured to the implants immediately following the procedure to preserve occlusal functionality and aesthetic appearance. The patient was provided with postoperative instructions and followed up regularly to assess the healing process and implant stability. The implant sites were allowed to heal for four months, with regular assessments of oral hygiene throughout the healing phase to maintain peri-implant health. After four months, the patient was recalled for the second-stage surgery, during which the implant sites were surgically exposed, and the cover screws were replaced with gingival formers.

Prosthetic phase

**Figure 3:**
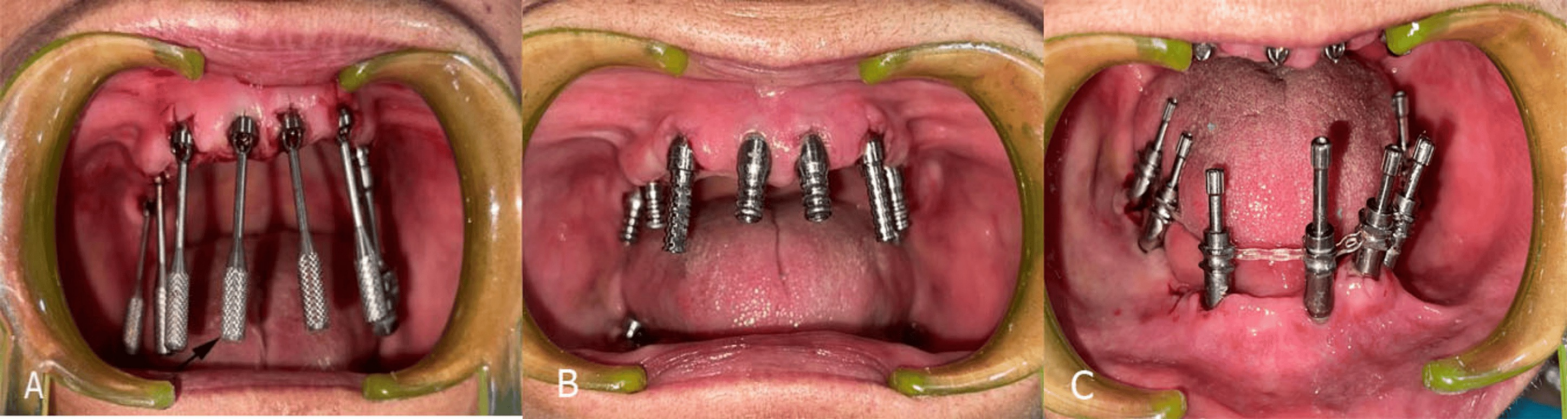
(A) Transmucosal abutments placed in the maxillary arch; (B) closed tray transfer copings in the maxillary arch; (C) splinted open tray transfer copings in the mandibular arch.

Fifteen days after the second stage of surgery, the patient was recalled for the final impression. The healing abutments were removed and replaced with open tray transfer copings in the mandibular arch, which were splinted using an elastomeric chain and an autopolymerizing resin (Pyrax, Roorkee, India). Closed-tray transfer copings were used for the maxillary arch (Figure 3).

A final single-step putty-wash impression was made using polyvinyl siloxane addition silicone (Pyrax, Roorkee, India). The impression was sent to the laboratory, where a master cast was poured using a type IV dental stone (Pyrax, Roorkee, India) with implant analogs. A verification jig was fabricated on the master cast using autopolymerizing resin and checked intraorally to ensure a passive fit and to confirm accurate implant positioning.

**Figure 4:**
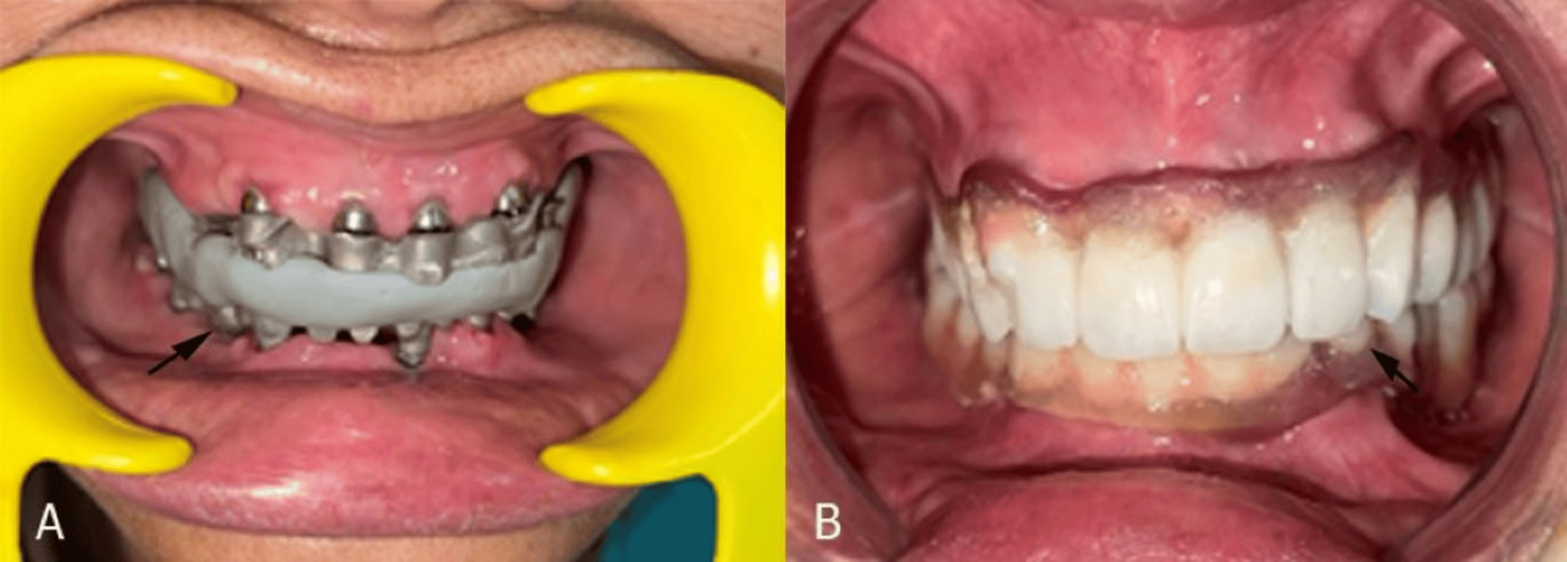
(A) Bite registration; (B) hybrid prosthesis cementation.

Subsequently, a metal framework was fabricated and assessed intraorally for precise, passive fit. After confirming the fit of the framework, a final interocclusal bite registration was performed using a condensation silicone material (Zhermack, Badia Polesine, Italy) with the metal framework in place (Figure 4A).

Shade selection was then carried out using the VITA Classical Shade Guide (VITA Zahnfabrik, Bad Säckingen, Germany), followed by an acrylic build-up. Selective occlusal adjustments were performed after the bisque trial; the prosthesis was subsequently glazed, and a trial denture was evaluated for esthetics and occlusion. The screw-retained hybrid metal framework was torqued to the implants at 30 Ncm, the screw access holes were sealed with Teflon tape (Chemours, Wilmington, DE) and composite resin, and occlusal adjustments were made to establish mutually protected occlusion. The final implant-supported prosthesis successfully restored both function and esthetics (Figure 4B). The patient was instructed to maintain oral hygiene and was scheduled for regular follow-up appointments, during which the necessary occlusal adjustments were made.

Post-rehabilitation

**Figure 5:**
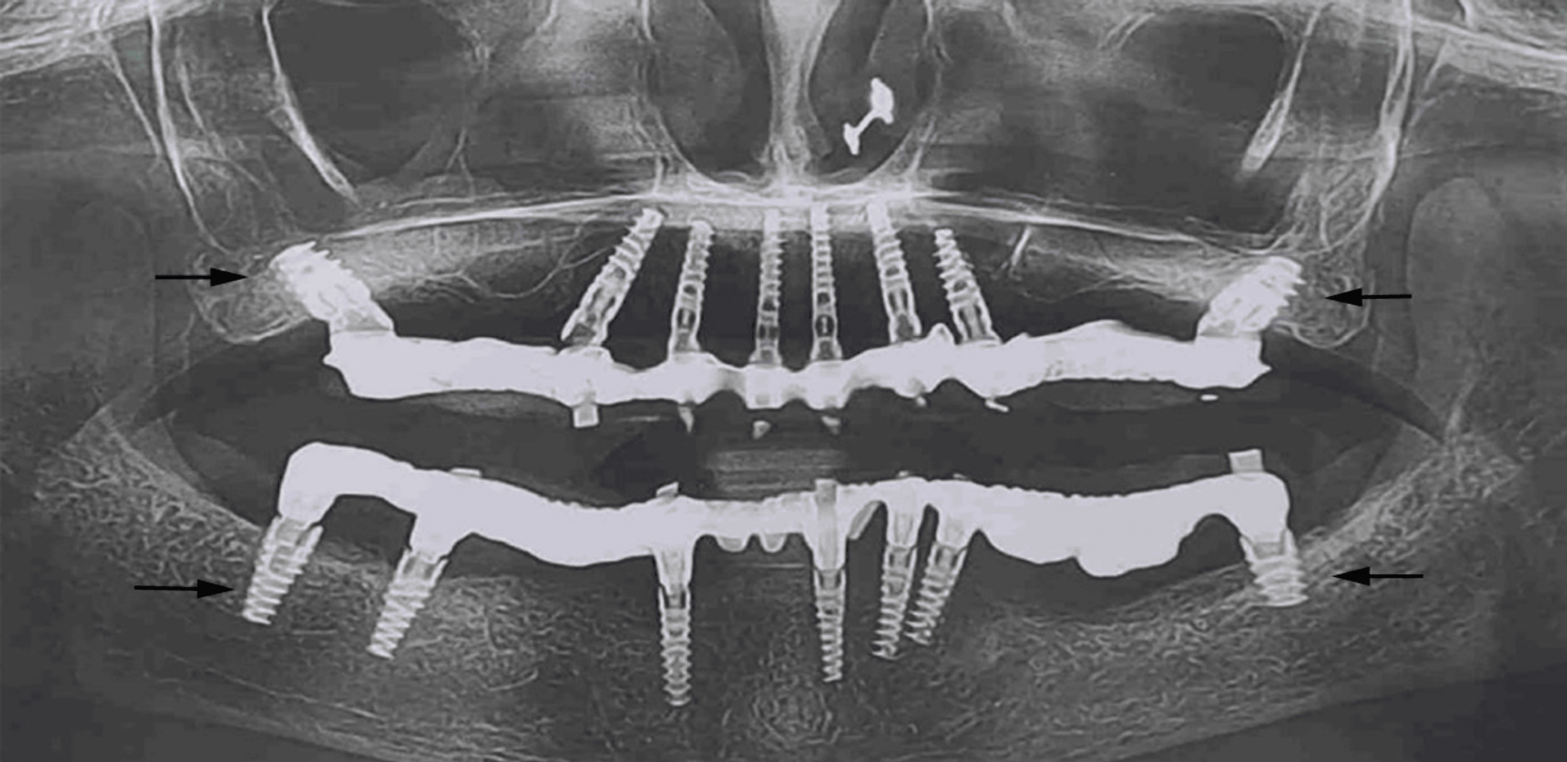
Post-rehabilitation orthopantomogram.

Post-rehabilitation instructions were given, and the patient was recalled for follow-up. The patient was evaluated 24 hours postoperatively, and a follow-up OPG was performed (Figure 5).

Minor adjustments were made as necessary. Routine follow-up clinical assessments after prosthetic delivery were conducted at one, three, and six months. The patient expressed satisfaction with esthetics and speech and significantly improved masticatory function. Her positive attitude and satisfaction with treatment contributed to a favorable prognosis and overall rehabilitation success.

## Discussion

The successful rehabilitation of a 75-year-old female patient using an implant-supported hybrid denture reflects both contemporary prosthodontic principles and advances in implantology. This case emphasizes the importance of addressing both functional and esthetic concerns, particularly in elderly patients with significant oral health challenges such as alveolar bone loss and residual root stumps. The use of a hybrid denture, in this case, allowed for predictable, cost-effective, and functionally stable outcomes, which is in line with recent studies in the literature.

A primary consideration in this case was the patient’s desire for a fixed prosthetic solution. As highlighted in the literature, elderly patients often prefer fixed restorations owing to enhanced masticatory function, esthetic improvements, and psychological satisfaction compared to removable dentures [4,6]. In this context, hybrid dentures, which combine the stability of fixed prostheses with the soft tissue support of removable ones, are highly beneficial [5]. They provide substantial lip and facial support, especially in cases where alveolar ridge resorption is prominent, which is consistent with Khatami and Smith’s observation that hybrid prostheses offer improved esthetics and function in such clinical scenarios [12].

Cylindrical and tapered implants are suitable for implant-supported hybrid prostheses. A systematic review highlighted that tapered implants exhibit a more favorable bone response than cylindrical implants [10]. Additionally, studies recommended the "All-on-6" concept for atrophied maxilla restoration, noting its higher success rate, reduced plaque accumulation, less crestal bone loss, and greater stability after 12 months compared to the "All-on-4" concept [13].

This case followed a delayed loading protocol, allowing for a four-month healing period after implant placement. This approach, based on Brånemark’s protocol, has been validated in multiple studies, showing higher success rates due to the complete osseointegration of implants before prosthetic loading [9,11]. Osseointegration is particularly critical in elderly patients, as factors such as decreased bone density and slower healing rates can affect the stability and long-term success of implants. Recent studies have shown that delayed loading remains an effective approach for improving implant longevity in patients with compromised bone quality [2,14].

The use of a verification jig during the prosthetic phase, in this case, ensured that the implants were accurately positioned, preventing any stress on the final prosthesis and promoting long-term stability. Verification jigs play a crucial role in complex cases involving multiple implants as they confirm the passive fit of the prosthetic framework and prevent any misalignment or mechanical complications [15]. In this case, the jig confirmed a passive fit, consistent with the existing literature that highlights the importance of accurate prosthesis fabrication to ensure implant survival.

A key point of success in this case was the patient’s positive attitude and compliance with the postoperative care and maintenance protocols. According to recent studies, patient compliance significantly affects the long-term success of implant-supported restorations. Maintaining oral hygiene and regular follow-ups, as provided in this case, are critical in preventing complications such as peri-implantitis, a common issue in elderly patients [2,5]. Furthermore, careful attention to occlusal adjustments during follow-up appointments helps maintain a mutually protected occlusion, ensuring the prosthesis's functional longevity and patient comfort [14].

## Conclusions

In conclusion, this case demonstrates the effectiveness of implant-supported hybrid dentures in the rehabilitation of elderly patients with partial edentulism. By following established protocols for implant placement, osseointegration, and prosthetic fabrication, optimal outcomes were achieved. Studies have confirmed that hybrid prostheses provide a versatile solution in complex cases, offering improved function, esthetics, and patient satisfaction while minimizing the risk of implant failure. This case underscores the importance of individualized treatment planning, precision in prosthetic design, and patient-centered care to ensure successful long-term outcomes.
